# Weak experiences sufficient for creating illusory figures that influence perception of actual lines

**DOI:** 10.1371/journal.pone.0175339

**Published:** 2017-04-18

**Authors:** Simon Hviid Del Pin, Kristian Sandberg, Bo Martin Bibby, Morten Overgaard

**Affiliations:** 1 Cognitive Neuroscience Research Unit, CFIN, Aarhus University, Aarhus, Denmark; 2 Consciousness Lab, Institute of Psychology, Jagiellonian University, Krakow, Poland; 3 Hammel Neurorehabilitation and Research Centre, Aarhus University Hospital, Hammel, Denmark; 4 Institute of Cognitive Neuroscience, University College London, London, United Kingdom; 5 Department of Biostatistics, Aarhus University, Aarhus, Denmark; Ecole Polytechnique Federale de Lausanne, SWITZERLAND

## Abstract

The question of whether conscious experience is best viewed as graded or dichotomous has received much scientific attention in recent years as the answer is relevant not only to models of consciousness, but also to the examination of neural markers of consciousness in patients and infants. Although some studies have found evidence of graded perception, it is unclear whether such perception is simply composed of individual stimulus features perceived in an all-or-none manner. Here, we examined whether the Kanizsa triangle (an illusory figure that is supposedly only perceived when all its parts are visible) has an impact on line length discrimination across four degrees of subjective visibility. We found that the presence of the Kanizsa triangle biases line length judgments (a phenomenon called the Ponzo illusion) when participants reported any experience (even a weak glimpse) of the stimulus. The results support the view that consciousness is a graded phenomenon. The strength of this support depends on the assumption that all parts of the illusory figure must be perceived for the illusion to work but this assumption is not resolved in the present literature. Currently, evidence can be found both for and against this notion.

## Introduction

Conscious experience is often assumed to be an all-or-none phenomenon that is either present or absent on a single trial basis in experimental settings (see for instance [1]), and this assumption is critical to central theories of consciousness [2,3]. Here, we present evidence supporting the notion that conscious experience is graded and that the gradedness is not caused by all-or-none perception of individual features, but instead that conscious experience is graded in a holistic sense.

In recent years, several experiments asking participants to rate their own conscious experience have found that consciousness appears graded rather than dichotomous (all-or-none). These results have been found using explicit introspective measures, confidence ratings, and post-decision wagering, using different experimental paradigms, perceptual modalities (visual and auditory), patient groups, and in combination with neuroimaging techniques by several different, independent research groups [4–13]. The typical finding has been that task accuracy varies in a graded manner as a function of awareness rating and that awareness ratings increase gradually as a function of stimulus intensity.

Although these findings appear stable and replicable, the interpretation is debated. According to one perspective, consciousness is dichotomous (all-or-none), and one proposal for reconciling the findings with this perspective is The Partial Awareness Hypothesis [14]. The hypothesis is that the perceptual system is hierarchically organized, and representations at different levels may be consciously accessed individually. These levels can be lower levels of (e.g.) vision such as energy or simple features as well as higher levels including concepts and words. From this perspective, a graded experience is one for which some, but not all, levels of representation are processed in an all-or-none manner. This view explains why participants in experiments are typically less correct, yet above chance level, in tasks for which they report experiences that are neither clear nor unconscious. More controversial, perhaps, the theory would expect a graded conscious experience to involve clearly experienced fragments of visual stimuli.

The Partial Awareness Hypothesis' view on mental representations is consistent with prominent theories of consciousness, such as the Neuronal Global Workspace Theory [2,15,16]. Here, a fundamental principle is consciousness being all-or-none, and this particular feature has been guiding search for neural markers of consciousness in infants [17] and in patients with so-called disorders of consciousness [18]. Support for the view of conscious experience as an all-or-none phenomenon has mainly come from observations of bimodal distributions of awareness ratings in the attentional blink and masking paradigms [19,20], and subsequently, an electrophysiological component, the P3 or P3a, has been observed to follow this all-or-none pattern [21].

The view that consciousness is “all-or-none” has been challenged in various ways–and, as a consequence, this interpretational framework as well. It has been proposed that the bimodal rating pattern observed in earlier studies could be the result of using reporting scales with only two points or a high number of points (e.g. 21 points with labels at the endpoints only [20]) resulting in a “smearing” of reports between endpoints [9]. Indeed, much more graded responses are observed when participants use a 4-point scale [9,11,22]. It has also been proposed that dichotomous results are a consequence of high level tasks (e.g. conceptual or mathematical questions) in masking paradigms [5,13], and the bimodal response pattern disappears in the attentional blink when the task difficulty is altered [6]. Common to all studies observing graded reports of awareness is that the mode of the rating distribution varies as a function of stimulus intensity, and each rating step is associated with a different accuracy level when each scale point is associated with a meaningful content (e.g. “a feeling that something was shown, but not characterized by any content”).

Whereas such arguments suggest alternative interpretations of dichotomous findings, they cannot rule out that the processing of single stimulus features explains intermediate levels of accuracy for weak perceptual experiences. If a weak experience implies processing of a single stimulus feature, accuracy will depend on whether this feature is diagnostic–if it can be used to distinguish between response options, accuracy is high; if not, accuracy is low. Thus, intermediate accuracy levels for weak experiences may be a result of high accuracy trials mixed with low accuracy trials.

Alternative interpretations argue that consciousness does not just appear graded in a number of experiments–consciousness is graded in itself. This view is apparent in, for example, The Radical Plasticity Thesis [23] and the Reorganization of Elementary Functions and CONsciousness model (REF-CON) (24). REF-CON proposes that a mental representation can be holistically degraded, e.g. more or less “clear”, and that such variations in clarity correspond to how cognitively accessible the given representation is, and, accordingly, it explains reduced levels of accuracy above base chance. Although The Partial Awareness Hypothesis and The Radical Plasticity Thesis/REF-CON all try to explain reports of gradual experience, they give different accounts on a central issue: Are vague experiences a matter of holistically reduced levels of clarity or a matter of fragmented conscious or unconscious components?

**Fig 1:**
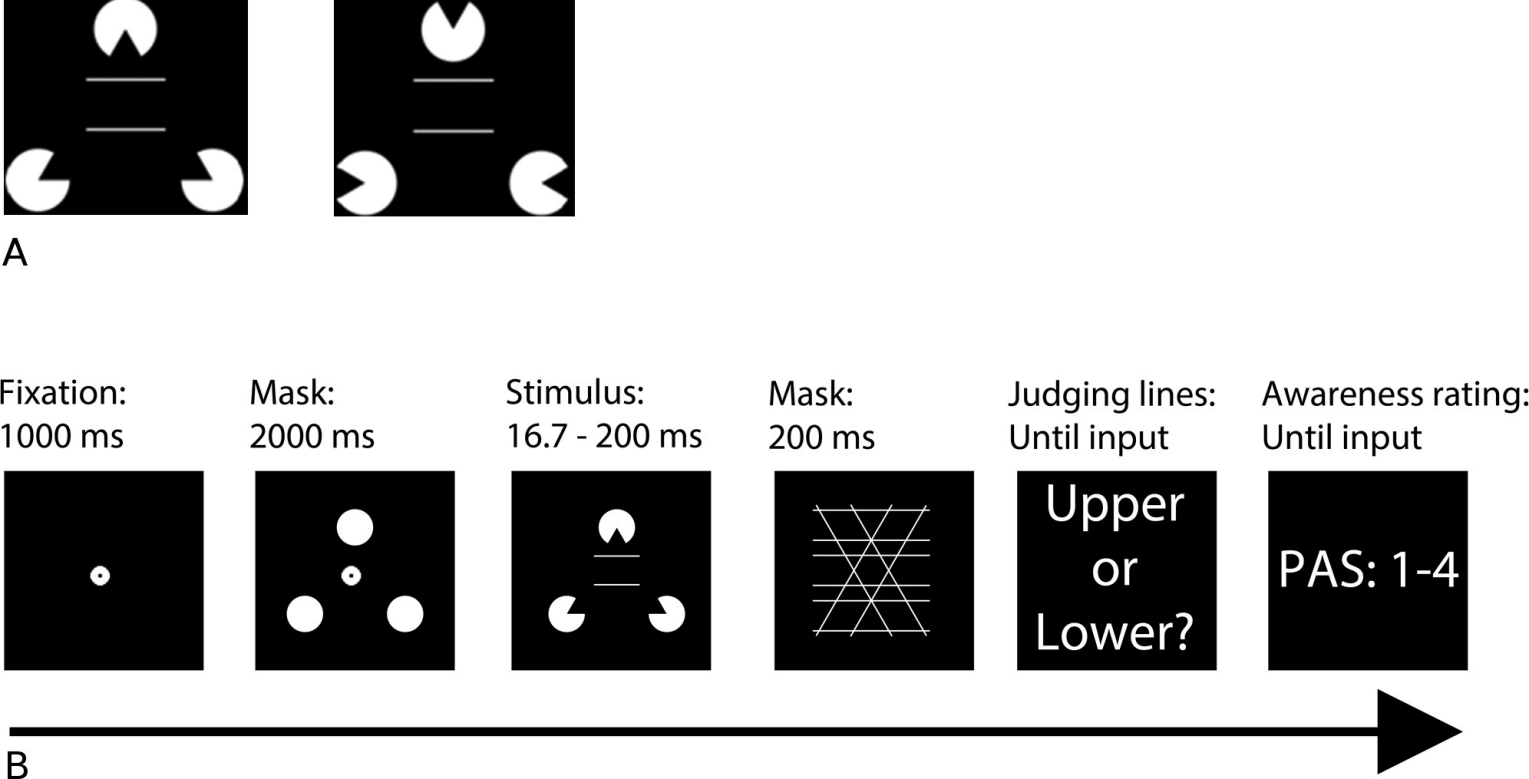
Stimuli and paradigm. A) Inducer configuration. Left: Inducers configured to induce perception of an illusory triangle (illusion condition). Right: Inducers configured to avoid inducing perception of an illusory triangle (control condition). B) Experimental paradigm. A fixation dot was displayed for 1000 ms and followed by an inducer forward mask consisting of 3 circles for 2000 ms. The figure extended 4.9 deg. horizontally to both sides from the fixation cross and 4.4 deg. Vertically. 2 lines (the lower line being kept constant at 3.9 deg.) surrounded by 3 inducers were then displayed for 17 to 200 ms. Inducers could be configured either as inducing an illusion or not (illusion condition shown). Next, a backward mask was displayed for 200 ms. Afterwards, the participant judged which line was the longest (upper or lower), and then rated her awareness on the 4-point perceptual awareness scale (PAS).

To investigate the two proposals, we created a paradigm wherein single stimulus fragments or features below the overall shape are not indicative. The paradigm used the illusory line segments of a Kanisza triangle [25] to bias the relative length estimates of two other lines, thus inducing the Ponzo illusion (Fig 1A) [26]. The underlying assumption is that the Kanizsa triangle is not perceived unconsciously [27], and does not appear when only a subset of inducers is perceived. We assume, in other words, that it is not possible to perceive the illusory stimulus features inducing the Ponzo illusion (i.e. the sides of the Kanizsa triangle) unless the shape is perceived as a whole (we will return to this assumption in the Discussion). If perception can be degraded as a whole, it can be hypothesised that the Kanizsa triangle induces a Ponzo illusion whenever participants have any experience of the stimulus. In contrast, if perception can be degraded by complete perception of single fragments or features only, it can be hypothesised that the Kanizsa triangle induces a Ponzo illusion only when the threshold of awareness is crossed, i.e. we should expect the illusion to have a sudden impact on accuracy when the stimulus is reported as completely clear (or possibly as almost clear). In other words, if participants have a weak experience of the illusory triangle as a whole, we predict a bias in the line discrimination task when intermediate awareness ratings are used (although the exact magnitude of the bias may be stronger when the experience is clearer). In contrast, if participants perceive only fragments of the inducers, and thus do not experience the triangle until they experience all inducers, we expect that the illusion does not bias line length discrimination when participants report weak experiences. In this way, we believe that the two proposals above will have opposing predictions for the outcome of line length discrimination.

We conducted a total of four experiments in which participants on each trial were presented with two lines and asked to judge which was longer. The line length of the upper line varied across trials and could be 15% shorter or longer than the lower line, or the lines could be of equal length. On all trials, the lines were surrounded by three inducers. The inducers were angled to induce the Kanizsa triangle illusion on half of the trials, and they were angled to avoid inducing the triangle illusion on the other half of the trials (Fig 1A). Stimuli were presented across a wide range of stimulus durations (16.7–200 ms) and were forward and backward masked (Fig 1B). The forward mask was used to avoid drawing attention to the inducers of the Kanizsa triangle. The backwards mask was to make the task more difficult and yield all possible PAS-ratings. Furthermore it was designed to disrupt the experience of the Kanizsa figure. On each trial, participants reported which line they judged to be longer (upper vs. lower) and how clear their experience was (on the Perceptual Awareness Scale, PAS [28]). This design allowed us to test for an effect of the presence of the illusion on the line comparison judgment for each PAS rating separately. The PAS rating of greatest interest was PAS2. Data for this rating allowed us examine whether the illusion was present when only a “weak glimpse” was perceived.

## Methods

Four experiments were conducted: a pilot experiment and three main experiments. The experimental paradigm was identical for the pilot experiment and Experiments 1–3 with the exceptions regarding stimulus durations, illusion display and participants mentioned below. The main goal of the pilot experiment was to examine if an effect of the illusion was present for each PAS rating overall and how it varied across stimulus durations. The goal of Experiment 1 was to establish the illusion effect clearly for PAS2 using the stimulus duration interval at which the pilot experiment had revealed the largest effect. Experiment 2 was a control experiment conducted with a higher number of participants to ensure that the illusion effect was also found for PAS2 at the stimulus intensities where this rating was used most frequently and thus that the effect was not just a special case observed only at high stimulus intensities. In the pilot experiment and in Experiment 1–2, a report bias was found that might indicate that the illusion was also present to a small degree in the control condition. Experiment 3 demonstrates that the illusion effect is also present for PAS2 when a bias-free illusion configuration is used.

### Participants

Participants were recruited from a university database in which people interested in participating in experiments have signed themselves up. They were contacted via mails announcing the experiments in year 2013–2014. All participants tested, completed the experiment. The inclusion criteria for the study was: age between 18–35 years, normal or corrected-to-normal visual acuity, and no use of psychiatric medication. There were no exclusion criteria. 16 healthy participants (11 females, mean age = 22.7 years, SD = 2.0 years, age range 18–25) participated in the pilot experiment. In Experiment 1, 16 healthy participants (7 females, mean age = 23.7, SD = 1.74 years, age range 20–27) participated. The number of participants were decided a priori as the pilot experiment indicated that for at least PAS 3 and 4, we could observe a significant effect of the illusion (p<0.001), and by focussing on the stimulus durations for which the effect size for PAS2 was largest (and leaving out the durations with little or no effect), we expected that this number would be enough to detect an effect statistically. In Experiment 2, 30 healthy participants (16 females, mean age = 22.3, SD = 2.3 years, age range 18–28) participated. A priori we expected a smaller effect here, and to diminish the risk of a type II error, we decided to increase the number of participants to 30, the limit dictated by our available resources in terms of time and money. All participants gave informed written consent and were paid 100 DKK to participate. The local ethics committee, The Central Denmark Region Committees on Health Research Ethics provided written confirmation that no ethical approval was required for the study according to Danish law, specifically Komitéloven §7 and §8.1.

### Stimuli and apparatus

A stimulus set was created with a control and an illusion condition (Fig 1B). Both stimuli consisted of two lines surrounded by 3 partial circles (Kanizsa illusion inducers). Stimuli were presented in white on a black background. For the control condition, the inducers were configured in a way that did not result in the perception of an illusory triangle, and perception of the perceived relative length of the lines was thus not influenced by the illusion in this condition. For the illusion condition, the inducers were configured in a way which yielded a Kanizsa triangle. The illusory line segments of the sides of the Kanizsa triangle were predicted to influence the perception of the two target lines in the centre of the stimulus display, i.e. creating a Ponzo illusion. The length of the upper line relative to the lower line varied across trials in a pseudorandom manner (3 possibilities: −15%, 0%, and +15%).

To run the experiment, custom Python code was executed in OpenSesame 0.27.2 ‘Frisky Freud’. Input was made via the native keyboard of a 14” laptop and recorded by OpenSesame. The display was on the laptop’s native screen (60hz, 1366 x 768 pixels in 16:9 format; brightness: 209.4 cd/m^2^). There was not a fixed distance to the screen, but the participants were asked to place themselves in a comfortable position (usually roughly 50 cm).

### Procedure

All experiments consisted of 18 blocks of 24 trials (432 trials in total). Each trial began with a fixation dot displayed for 1000 ms (Fig 1B). The dot was followed by a forward mask consisting of 3 circles displayed for 2000 ms. Next, the target horizontal lines were displayed surrounded by 3 Kanizsa illusion inducers. The stimulus duration varied across trials in a pseudorandom manner within experiments as well as across experiments. In the pilot experiment, 16.7, 50, 100, and 200 ms were used, and. In Experiment 1, 16.7, 50, 100, 150, and 200 ms were used. The three latter stimulus durations were used in 108 trials each and the two first were used only in 54 trials each. In Experiment 2, 16.7, 50, 67, 83 100, and 200 ms were used. Stimulus durations 67, 83, and 100 ms were used in 108 trials each and the remaining were used only in 36 trials each. The pilot experiment thus examined a wide range of stimulus durations, whereas Experiments 1–2 examined long and intermediate stimulus durations in greater detail. All experiments used the same overall range of stimulus durations to ensure consistent use of awareness ratings.

The stimulus display was followed by a backward mask displayed 200 ms. The mask was shaped to ensure that both the horizontal target lines as well as the illusory Kanizsa and the inducer lines were masked. The participant then indicated which line was judged to be the longest (forced choice). Finally, participants rated their experience on the 4-point Perceptual Awareness Scale (PAS) (28), see Table 1). The response options were: “1: No experience”, “2: A weak experience”, “3: An almost clear experience”, and “4: A clear experience”. Prior to the actual experiment, each participant would have 24 trials in which they were trained to use the PAS. The training consisted of a one-on-one session between the participant and the experiment leader. In order to encourage consistent PAS usage, participants were asked open ended questions such as “I noticed that you pressed 2, why did you choose that?” or “What would you have rated if you had seen at least one of the lines clearly?”. After the training each participant was asked whether they felt comfortable using the scale before moving on to the main experiment. Participants were instructed to rate the lines and told that the circles would be there and that they could ignore them as they were not part of the task. Some participants expressed that they were useful for navigating their eyes to where the lines were and they were told that this was fine as well.

**Table 1:** The Perceptual Awareness Scale (PAS). Scale steps and descriptions.

Label	Description (from Ramsøy & Overgaard, 2004)
1: No experience	No impression of the stimulus. All answers are seen as mere guesses
2: A weak experience	A feeling that something has been shown. Not characterised by any content, and this cannot be specified any further
3: An almost clear experience	Ambiguous experience of the stimulus. Some stimulus aspects are experienced more vividly than others. A feeling of almost being certain about one’s answer
4: A clear experience	Non-ambiguous experience of the stimulus. No doubt in one’s answer

Each experiment lasted approximately 1 hour including instructions and self-paced breaks. After the experiment participants were debriefed and comments on the experience of illusion as well as the usage of the PAS were noted.

### Analysis of data

We created a mixed logistic regression model with line length judgement as dependent variable (0: lower longest, 1: upper longest) and illusion inducer configuration (illusion or control), line length (upper shorter than, equal to, or longer than lower), and awareness rating (PAS rating 1–4) as independent variables. Participant was added as a random effect. The effect of illusion was assessed based on a chi-square test comparing the deviance from this model with that from an identical mixed logistic regression model just without illusion as an independent variable. This was subsequently repeated for each PAS rating. A p-value below .05 was interpreted as providing evidence for that an illusion effect was present.

## Results

A pilot experiment with 16 participants is reported in the Supplemental Material available online. In summary, this experiment used a wide range of stimulus durations (16.7–200 ms) and showed a highly significant effect of illusion inducer configuration (illusion vs. control) on line length judgments. This effect was present when participants described their experiences as “almost clear” (PAS3) and “clear” (PAS4) (Chi^2^ (3) > = 66.4, p < .001), but it was unclear whether the effect was also present when participants reported to have “a weak experience” (PAS2) only (Chi^2^ (3) = 6.6, p = 0.09). Exploratory analyses revealed that the effect of illusion configuration for PAS2 appeared the largest for relatively high stimulus durations (100–200 ms) although PAS2 was used more frequently at lower stimulus durations (below 100 ms). In Experiment 1, 16 participants were tested using a large number of trials in the stimulus duration interval for which the largest effect for PAS2 was expected: 100, 150, and 200 ms. In Experiment 2, 30 participants were tested using a large number of trials in the interval for which PAS2 ratings were expected to be used most frequently: 67, 83, and 100 ms. S6 Fig. in the Supplemental Material shows the modelled likelihood for participants using each of the four PAS ratings. Overall, the distributions of PAS ratings were highly similar across illusion and control conditions in all four experiments.

### Experiment 1

**Fig 2:**
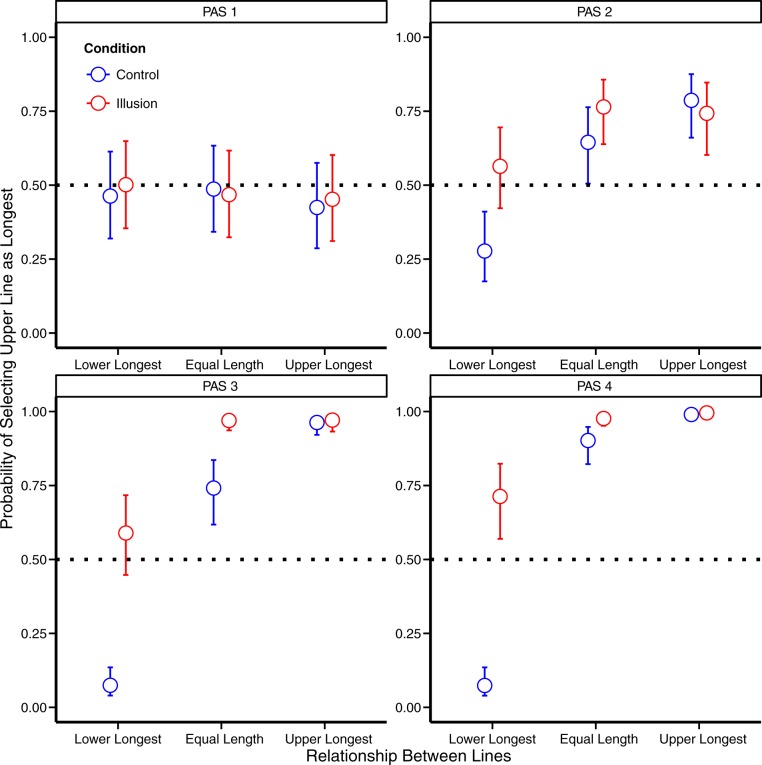
Probability of categorising the upper line as the longest (Experiment 1). Probabilities are reported for each awareness rating and for each line length condition. When participants (n = 16) rated “no experience” (PAS1), they did not perform statistically different from chance level. As awareness increased (PAS2-4), the probability of correct classification of lower/upper line longest conditions increased, and a bias for reporting the upper line as the longest was seen when the Kanizsa illusion was present. The bias was most clearly seen when the lower line was the longest and when the lines were equally long, possibly due to a ceiling effect when the upper line was longer. Error bars represent 95% Confidence intervals.

A chi-squared hypothesis test showed a clear overall effect of illusion (Chi^2^ (12) = 580.1, p < .001, Fig 2). For PAS 1, however, there was no significant effect of illusion (Chi^2^(3) = 1.4, p = .70) and when the lines were of equal length, the odds ratio for choosing the upper line as the longest between illusion and control was 1.03 (95%-CI: 0.73;1.46) which implies that there was no significant difference when participants compared equal lines under the two conditions. For PAS 2–4 there was a clearly significant effect of illusion (for all tests, Chi^2^(3) > 51.6, p < .001). The estimates of the odds ratios for choosing the upper line as the longest between illusion and control for all three ratings (PAS 2: 1.84 (95%-CI: 1.22;2.78), PAS 3: 11.21 (95%-CI: 6.15;20.45), PAS 4: 4.42 (95%-CI: 2.26;8.64)) indicate that participants reported the upper line to be longest significantly more under the illusion condition when comparing objectively equal lines. When comparing objective performance (i.e. how participants rated different lines under the control condition only–see supplementary for information about calculations), participants performed at chance level for PAS 1: 48.12% (95%-CI: 40.86;55.45%) for all other ratings participants, participants performed significantly better than chance (PAS 2: 71.08% (95%-CI: 64.87;76.60%), PAS 3: 92.69% (95%-CI: 89.72;94.85%), PAS 4: 96.55% (95%-CI: 94.90;97.68%))

Taken together, the analyses showed that taking into account whether the Kanizsa illusion was presented did not influence length comparison response on a trial by trial basis when participants report PAS1, i.e.”no experience”. In contrast, when participants reported vague or clearer experiences (PAS2-4), the illusion influenced the length comparison response. The illusory contours of the Kanizsa triangle thus had an impact on the magnitude of the Ponzo illusion even when participants reported seeing only a weak glimpse of the stimulus. This result is unlikely if the report of a “weak glimpse” was a result of participants perceiving only individual features or fragments of the illusion inducers.

### Experiment 2

**Fig 3:**
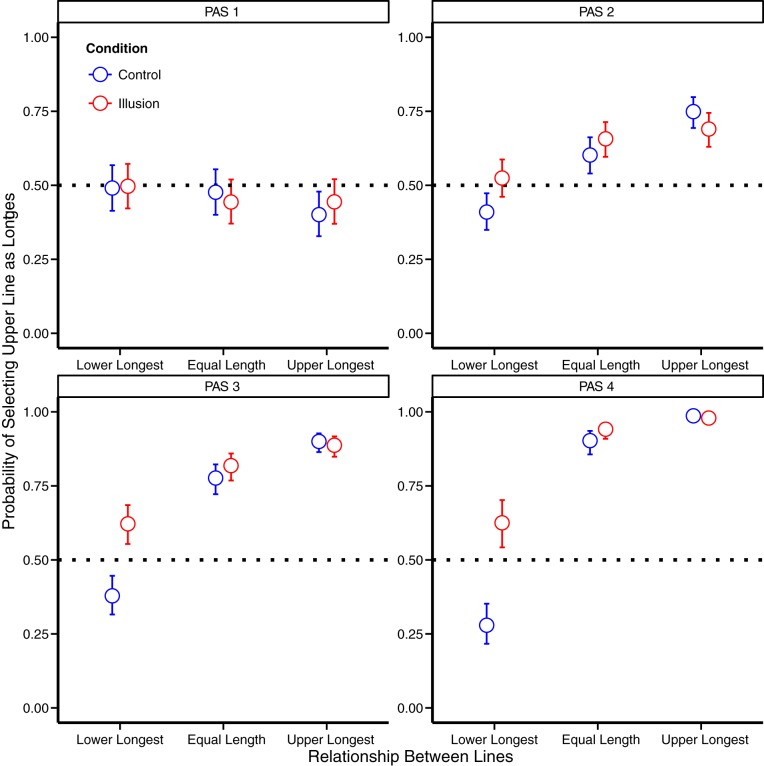
Probability of categorising the upper line as the longest (Experiment 2). Probabilities are reported for each awareness rating and for each line length condition. When participants (n = 30) rated “no experience” (PAS1), they did not perform statistically different from chance level. As awareness increased (PAS2-4), the probability of correct classification of lower/upper line longest conditions increased and a bias for reporting the upper line as the longest was seen when the Kanizsa illusion was present. The bias was most clearly seen when the lower line was the longest and when the lines were equally long, possibly due to a ceiling effect when the upper line was longer. Error bars represent 95% Confidence intervals.

A chi-squared hypothesis test showed a clear overall effect of illusion longest (Chi^2^ (12) = 171.6, p < .001, Fig 3). For PAS 1, there was no significant effect of illusion (Chi^2^ (3) = 1.4, p = .45)) and when the lines were of equal length, the estimate of the odds ratio for choosing the upper line as the longest between illusion and control was 0.84 (95%-CI: 0.61;1.16) which implies that there was no significant difference when participants compared equal lines under the two conditions. For PAS 2–4 there was a clearly significant effect of illusion (for all tests, Chi^2^ (3) > 35.5, p < .001) The estimates of the difference in odds for choosing the upper line as the longest between illusion and control for all three ratings (PAS 2: 1.29 (95%-CI: 1.05;1.57), PAS 3: 1.27 (95%-CI: 0.94;1.70), PAS 4: 1.56 (95%-CI: 0.88;2.78)) indicate that participants reported the upper line to be longest more under the illusion condition when comparing objectively equal lines for PAS 2 but not significantly so for PAS 3–4. One can notice that the estimates indicate a similar effect to PAS 2 and Experiment 1, but that the confidence intervals are wider. When comparing objective performance, participants performed at chance level for PAS 1: 47.63% (95%-CI: 41.78;53.55%). For all other ratings participants, participants again performed significantly better than chance (PAS 2: 66.19% (95%-CI: 61.65;70.45%), PAS 3: 76.95% (95%-CI: 72.88;80.56%), PAS 4: 91.32% (95%-CI: 88.77;93.34%))

The analyses showed that taking into account whether the Kanizsa illusion was presented did not influence the direction pressed on a trial by trial basis when participants reported ‘no experience’. When participants reported vague or clearer experiences (PAS2-4), the illusion influenced the direction. In other words, the illusory contours of the Kanizsa triangle again had an impact on the magnitude of the Ponzo illusion when participants reported seeing only a weak glimpse of the stimulus. This result is consistent with the results of experiment 1, but would be unexpected if the report of a “weak glimpse” was a result of participants perceiving only individual features or fragments of the illusion inducers.

It may be noted that a bias to report the upper line to be longer was also present in the control condition. In the Supplementary Material, we report the results of a bias-free paradigm (Experiment 3). The results of this experiment are qualitatively identical to those of Experiments 1–2 thus ruling out the bias as a potential confound. For readers preferring model comparison by the Akaike information criterion (AIC), the Supplementary Material contains a table of the AIC-values for each of the models reported in this paper (S1 Table). For all experiments, the simple model has the lowest AIC-value for PAS 1, whereas the full model the lowest AIC-value for PAS 2–4. Model selection with AIC would thus generally agree with the conclusions reported here, with the one exception that for PAS 2 in the pilot, the full model had the lowest AIC-value, while the simple model was not rejected by p-values (p = 0.09).

## General discussion

Understanding whether conscious experiences are graded or dichotomous is relevant to theoretical discussions on the nature of consciousness and to all attempts to interpret the conscious state of someone who is not verbally responding, be it an infant, a patient or a non-human animal. Therefore, we investigated the nature of representations at four distinct categories of experience. Our results across three experiments showed that a holistic visual illusion influenced participants when they reported having any (even vague) experiences of the presented stimuli. The Partial Awareness hypothesis posits that participants have a clear representation of some aspects while other aspects are not represented at all when their experiences are vague: “[I]*ntermediate situations* [i.e. vague experiences] *can also be explained as partial awareness situations and thus reflect access to different levels of representation (e*.*g*. *access to low-level features without access to object identity)*” [14]. This hypothesis appears incompatible with the present results as under the assumption that full perception of parts of the stimulus, or early stages of processing (e.g. perception of the amount of visual energy), cannot induce the illusion–only perception of the full stimulus can. The conclusion of the experiment depends on two assumptions: 1) That the Kanizsa illusion does not have an effect on behaviour when perceived unconsciously (for a short review, generally supporting this assumption, see [29]). And 2) that the illusion is only perceived when all inducers are perceived.

Since we collected our data, two papers relevant to the first assumption, and reaching opposite conclusions, have been published. In one paper a special kind of mask was applied alternating the stimulus with a randomly generated mask of inducer shapes with a total display time of 960 ms. In this setup, participants could distinguish an illusory diamond from an illusory square better than chance, while not being significantly better than chance to report properties of the inducers [29]. In the other paper, the performance of distinguishing the orientation of an actual low contrast triangle and a Kanizsa triangle was compared [30]. Here the conclusion was that the participants could significantly distinguish the orientation of the low contrast triangles but not the orientation of Kanizsa triangles. In fact they obtain Bayesian evidence (albeit weak) against that people can perceive Kanizsa figures unconsciously. Given the state of the art, we acknowledge that we currently cannot rule out the possibility that participants could be influenced by the illusion even though they did not perceive the inducers. Future studies could examine the impact of this potential confound by asking participants to report directly on the perceived clarity of the inducers instead of the horizontal lines in the stimulus display. In the present study, the participants were not told about the Kanizsa triangle before the experiment to minimize the risk of them inferring the influence of the Ponzo illusion, which might affect their response behaviour

Relevant to the second assumption, some evidence exists that two inducers are enough to generate an illusory contour. Gillam and Marlow [31], for instance, found that while a display of four inducers (arranged as a square) generated a strong illusion, a display of only two neighbouring inducers also generated an illusion, albeit a weaker one. Therefore, it cannot be ruled out entirely that weak glimpses could be explained by clear perception of neighbouring inducers causing a bias in the perceived length of the horizontal lines on one end only and thus biasing length judgments to a slightly lesser extent than is the case when all inducers are perceived. Future studies could control for this potential confound by using illusory displays with only two inducers. It may be noted that the expected illusion size is much smaller than in the full display used in the experiments presented here since only one illusory contour is used and this one contour is expected to be weaker. Such a study would thus require a relatively large number of participants (the exact number is difficult to estimate).

The results of the study do not provide direct support for the Partial Awareness hypothesis as they are only compatible with this hypothesis if the assumptions listed above have led to potential confounds. Assuming the potential confounds are not significant, the results nevertheless fit well with other parts of the literature hypothesising that consciousness is graded. One example is the radical plasticity thesis, positing that information processing is graded and continuous, taking place over many interconnected modules consisting of processing units and thus explicitly assumes that representations are graded, dynamic, active, and constantly causally efficacious [23]. Moreover, Tononi & Koch [32] posit in their Integrated information theory that consciousness is a completely graded fundamental property, possessable by various physical systems, even systems much simpler than the human brain. The results also fit with the REF-CON model according to which subjective experience is a graded phenomenon that fundamentally relates to how and to which degree information is available for (certain kinds of) action [24,33].

If the potential confounds are not significant, the results suggest that weak conscious experiences are not fragments or partial elements that are pieced together as the graded representation becomes more conscious. Rather, they suggest that graded experiences are holistically “unclear”–i.e. that the property of being a “holistic representation” is preserved at all levels of degradation. Future modes of investigation can focus on specific features of stimuli and investigate whether these are rated gradually or all-or-none. Another possible approach can be similar to the present experiment, in which we target specific levels of representations.

The view of consciousness as all-or-none has been guiding studies examining markers of consciousness in infants [17] and patients [18], and accordingly the studies have searched specifically for all-or-none neural components. While we do not question the general findings of these studies, the current results could be taken as support for the notion that graded neural components may be even more predictive as markers of consciousness. This is consistent with recent studies showing that the ability to predict the contents and clarity of conscious experience from MEG data peaks at the graded VAN, i.e. before the all-or-none P3a [4,34].

Taken together, we believe that the current study along with other recent studies provide some support for the notion that consciousness is a graded phenomenon, and that mental representations are holistic rather than all-or-none fragments, yet we emphasise that there are still a few potential confounds that could not be addressed in the four experiments presented here, but which will need to be addressed before strong evidence against an all-or-none theory of consciousness can be provided. The paradigm presented here provides a potentially useful framework for how these next studies may be conducted.

## Supporting information

S1 Fig Probability of categorising the upper line as the longest (Pilot Experiment). Probabilities are reported for each awareness rating and for each line length condition. When participants (n = 16) rated “no experience” (PAS1), they did not perform statistically different from chance level. As awareness increased (PAS2-4), the probability of correct classification of lower/upper line longest conditions increased. For PAS3-4, a bias for reporting the upper line as the longest was seen when the Kanizsa illusion was present whereas the results were inconclusive for PAS2. The bias was most clearly seen when the lower line was the longest and when the lines were equally long, possibly due to a ceiling effect when the upper line was longer. Error bars represent 95% Confidence intervals.
(EPS)

S2 Fig Exploratory analysis of illusion effect across stimulus durations (pilot experiment). A) Probability of using the PAS2 rating on a trial as a function of stimulus duration (n = 16). B) Bias for reporting the upper line as longer in the illusion condition (compared to control condition) is plotted as a function of stimulus duration for PAS2 ratings (n = 16). The dotted line at 0.5 indicates no effect of illusion. The illusion effect appears to increase across stimulus durations but so does the error bars (indicating 95% confidence intervals) due to the lower proportion of PAS2 ratings for higher stimulus durations.
(EPS)

S3 Fig Inducer configurations (Experiment 3). Left side: Inducers configured to induce perception of an illusory triangle in either the upper or lower visual field (illusion condition). The lines would always be presented in the illusory triangle. Right side: Inducers configured to avoid inducing perception of an illusory triangle (control condition). The lines could be presented either in the upper–or lower part of the visual field. All displayed line sets have equal lengths.
(TIF)

S4 Fig Probability of categorising the upper line as the longest (Experiment 3, Upper Triangle). Probabilities are reported for each awareness rating and for each line length condition. When participants (n = 16) rated “no experience” (PAS1), they did not perform statistically different from chance level. As awareness increased (PAS2-4), the probability of correct classification of lower/upper line longest conditions increased and a bias for reporting the upper line as the longest was seen when the Kanizsa illusion was present. The bias was most clearly seen when the lower line was the longest and when the lines were equally long, possibly due to a ceiling effect when the upper line was longer. Error bars represent 95% Confidence intervals.
(TIF)

S5 Fig Probability of categorising the upper line as the longest (Experiment 3, Lower Triangle). Probabilities are reported for each awareness rating and for each line length condition. When participants (n = 16) rated “no experience” (PAS1), they did not perform statistically different from chance level. As awareness increased (PAS2-4), the probability of correct classification of lower/upper line longest conditions increased and a bias for reporting the upper line as the longest was seen when the Kanizsa illusion was present. The bias was most clearly seen when the lower line was the longest and when the lines were equally long, possibly due to a ceiling effect when the upper line was longer. Error bars represent 95% Confidence intervals.
(TIF)

S6 Fig Modelled probability of using each PAS-rating. How likely were participants (N:Pilot = 16;Experiment 1 = 16;Experiment 2 = 30; Experiment 3 = 16) to use each PAS-rating? PAS-rating under the Control and Illusion condition generally appeared to be comparable. Error bars represent 95% Confidence intervals.
(TIF)

S1 Table AIC-values for the Simple–and Full models for each PAS-rating, each experiment. The Full model includes whether the illusion was presented or not. The lowest value for each model is marked with bold. A lower AIC-value for the full model indicates that the illusion influenced participant’s line judgement in a measurable way. For all experiments, the simple model is preferred for PAS 1, whereas the full model is preferred for PAS 2–4.
(DOC)
