# Supplementary figures and images for: Weak experiences sufficient for creating illusory figures that influence perception of actual lines
Source: PLoS One. 2017 Apr 18;12(4):e0175339. doi: 10.1371/journal.pone.0175339 (PMC5395153; doi:10.1371/journal.pone.0175339)

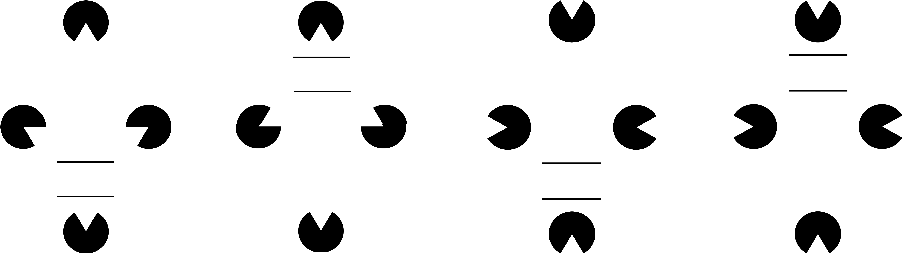

Supplement: S3 Fig — Left side: Inducers configured to induce perception of an illusory triangle in either the upper or lower visual field (illusion condition). The lines would always be presented in the illusory triangle. Right side: Inducers configured to avoid inducing perception of an illusory triangle (control condition). The lines could be presented either in the upper–or lower part of the visual field. All displayed line sets have equal lengths. (TIF) [file pone.0175339.s003.tif]

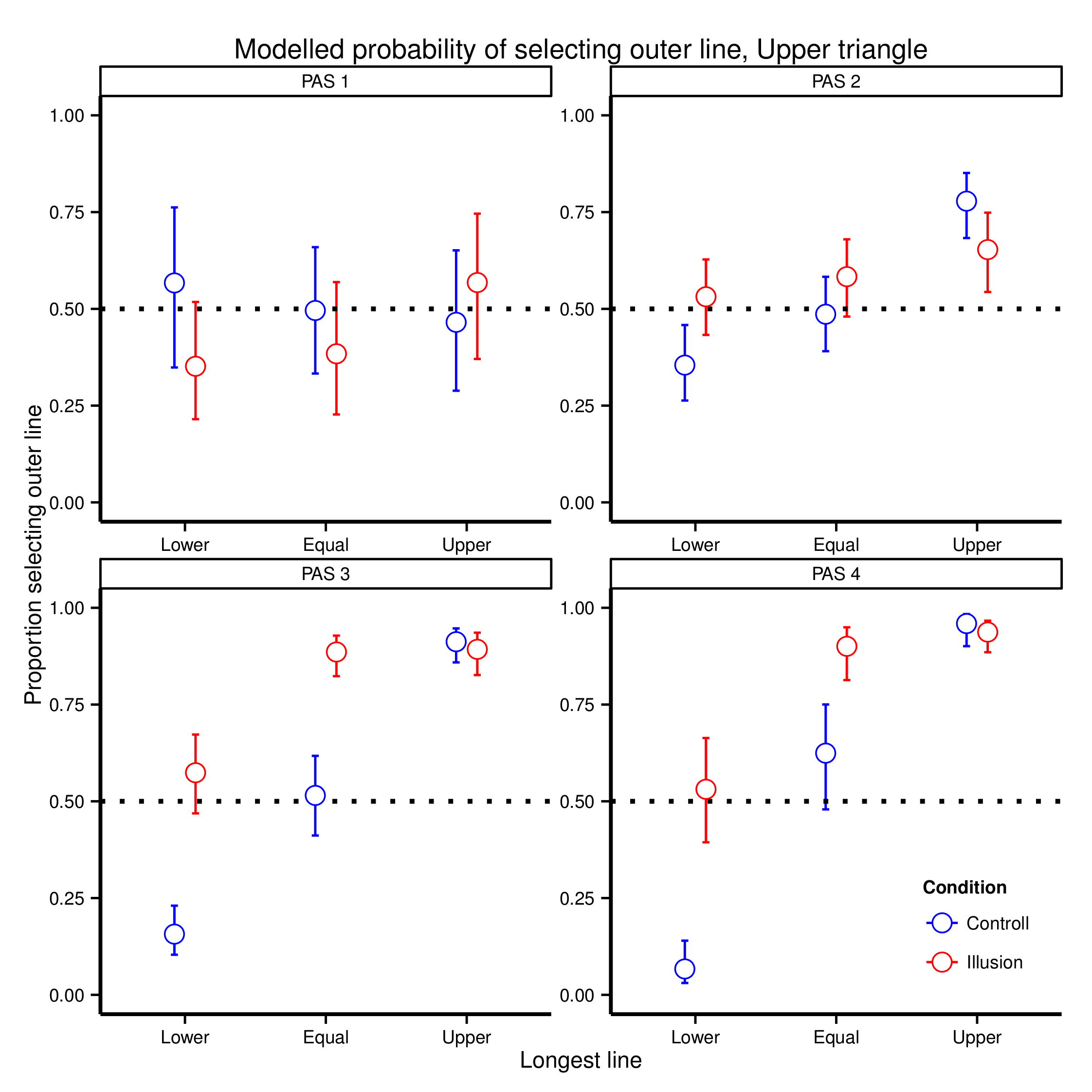

Supplement: S4 Fig — Probabilities are reported for each awareness rating and for each line length condition. When participants (n = 16) rated “no experience” (PAS1), they did not perform statistically different from chance level. As awareness increased (PAS2-4), the probability of correct classification of lower/upper line longest conditions increased and a bias for reporting the upper line as the longest was seen when the Kanizsa illusion was present. The bias was most clearly seen when the lower line was the longest and when the lines were equally long, possibly due to a ceiling effect when the upper line was longer. Error bars represent 95% Confidence intervals. (TIF) [file pone.0175339.s004.tif]

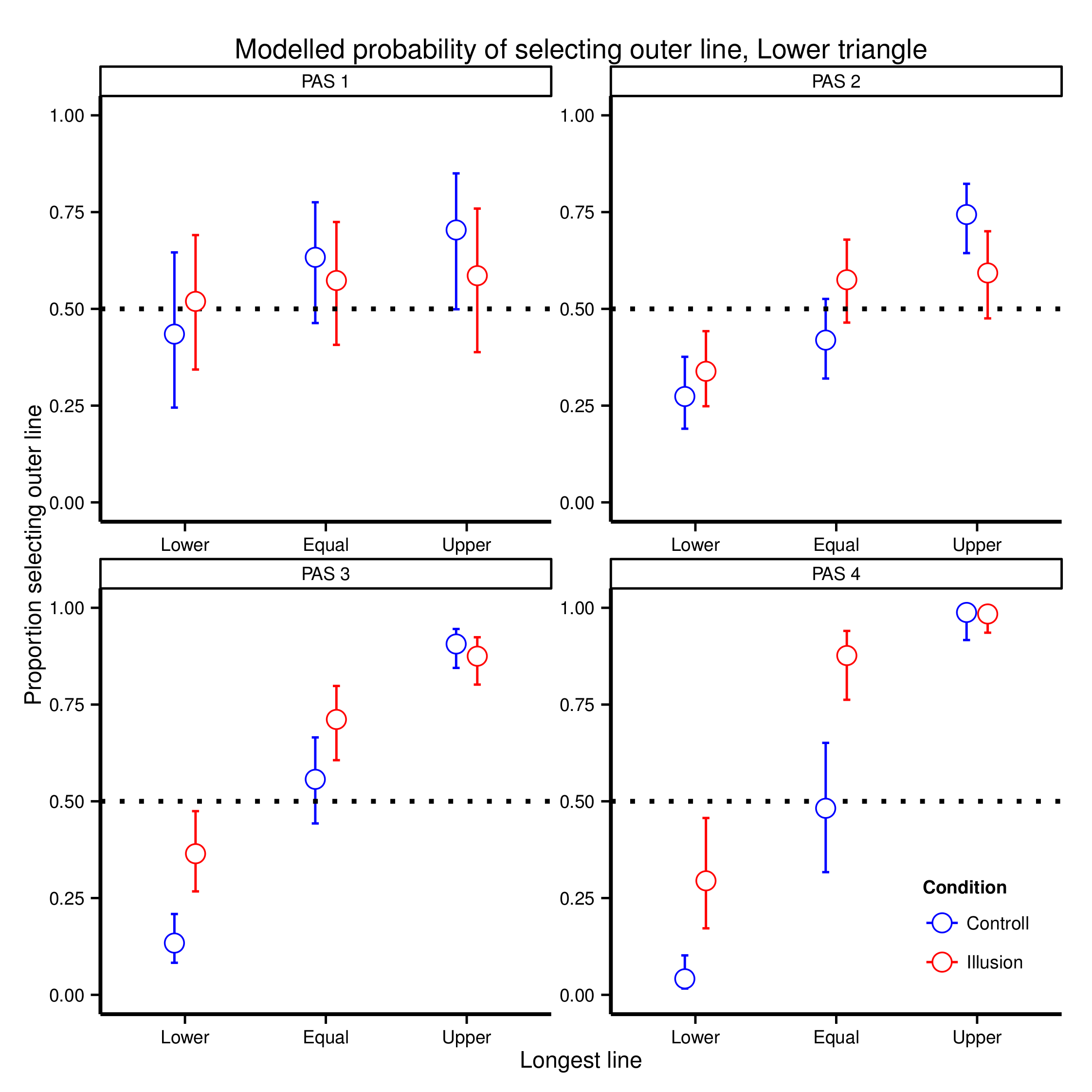

Supplement: S5 Fig — Probabilities are reported for each awareness rating and for each line length condition. When participants (n = 16) rated “no experience” (PAS1), they did not perform statistically different from chance level. As awareness increased (PAS2-4), the probability of correct classification of lower/upper line longest conditions increased and a bias for reporting the upper line as the longest was seen when the Kanizsa illusion was present. The bias was most clearly seen when the lower line was the longest and when the lines were equally long, possibly due to a ceiling effect when the upper line was longer. Error bars represent 95% Confidence intervals. (TIF) [file pone.0175339.s005.tif]

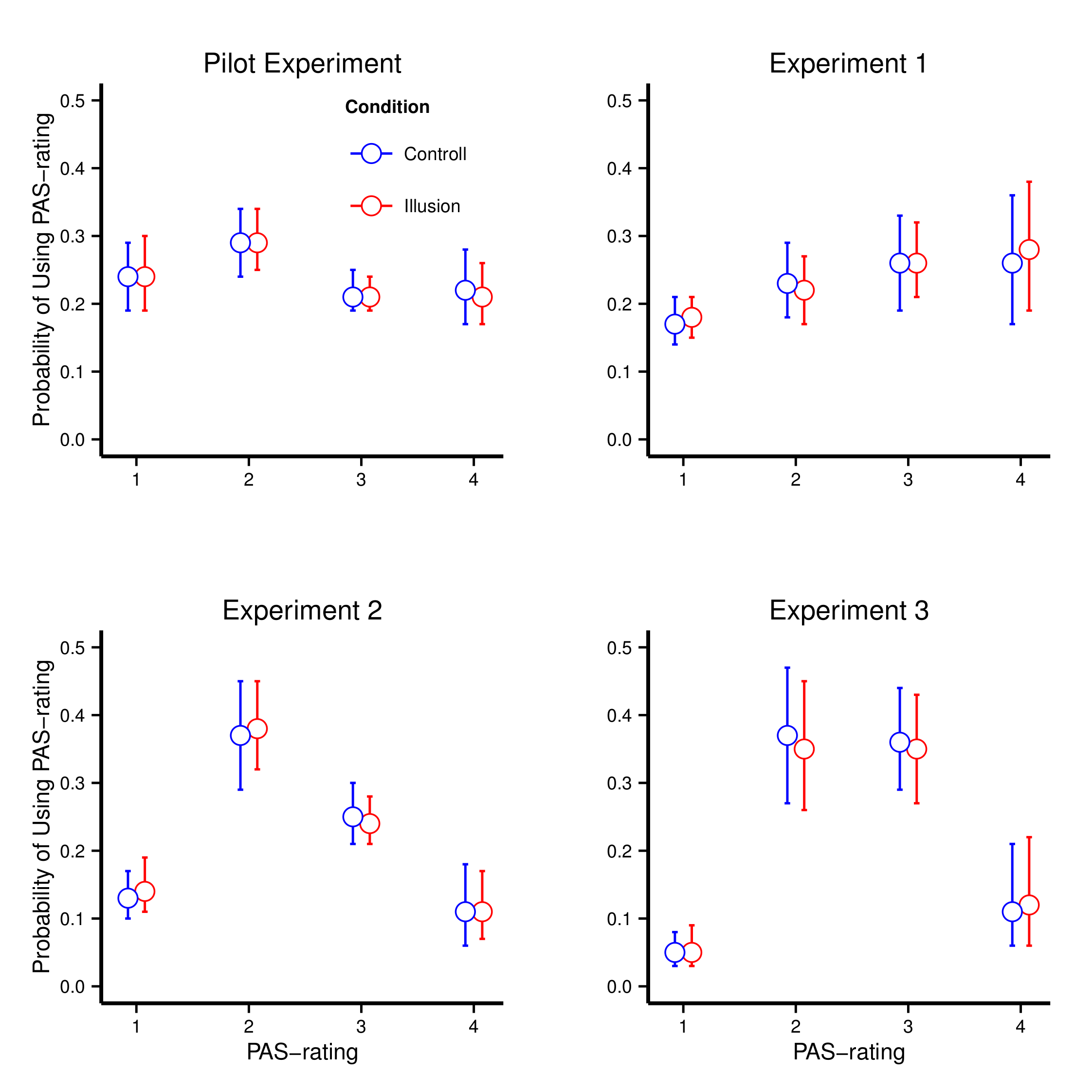

Supplement: S6 Fig — How likely were participants (N:Pilot = 16;Experiment 1 = 16;Experiment 2 = 30; Experiment 3 = 16) to use each PAS-rating? PAS-rating under the Control and Illusion condition generally appeared to be comparable. Error bars represent 95% Confidence intervals. (TIF) [file pone.0175339.s006.tif]
