# Supplementary material for: Weak experiences sufficient for creating illusory figures that influence perception of actual lines
Source: PLoS One. 2017 Apr 18;12(4):e0175339. doi: 10.1371/journal.pone.0175339 (PMC5395153; doi:10.1371/journal.pone.0175339)
Supplement: S1 Table — The Full model includes whether the illusion was presented or not. The lowest value for each model is marked with bold. A lower AIC-value for the full model indicates that the illusion influenced participant’s line judgement in a measurable way. For all experiments, the simple model is preferred for PAS 1, whereas the full model is preferred for PAS 2–4. (DOC) [file pone.0175339.s007.doc]

| **PAS-rating/Model** | **Pilot AIC** | **Exp. 1 AIC** | **Exp. 2 AIC** | **Exp. 3 AIC**  **(Upper)** | **Exp. 3 AIC**  **(Lower)** |
| --- | --- | --- | --- | --- | --- |
| PAS 1 Simple (4 Df) | **2105.2** | **1162.3** | **2605.1** | **285** | **300.7** |
| PAS 1 Full (7 Df) | 2109.6 | 1166.9 | 2608.4 | 285.9 | 304.2 |
| PAS 2 Simple (4 Df) | 2684.9 | 1946.9 | 6348.5 | 1353.8 | 1475.1 |
| PAS 2 Full (7 Df) | **2684.4** | **1901.4** | **6319.0** | **1349.5** | **1463.6** |
| PAS 3 Simple (4 Df) | 1428.1 | 1680.8 | 3356.6 | 1337.4 | 1340.1 |
| PAS 3 Full (7 Df) | **1367.7** | **1392.1** | **3293.8** | **1227.2** | **1323** |
| PAS 4 Simple (4 Df) | 666.8 | 1021 | 1330.6 | 679.7 | 559.1 |
| PAS 4 Full (7 Df) | **558.4** | **826.7** | **1272.6** | **600** | **484.5** |

**S1 Table. AIC-values for the Simple – and Full models for each PAS-rating, each experiment.** The Full model includes whether the illusion was presented or not. The lowest value for each model is marked with bold. A lower AIC-value for the full model indicates that the illusion influenced participant’s line judgement in a measurable way. For all experiments, the simple model is preferred for PAS 1, whereas the full model is preferred for PAS 2-4.
